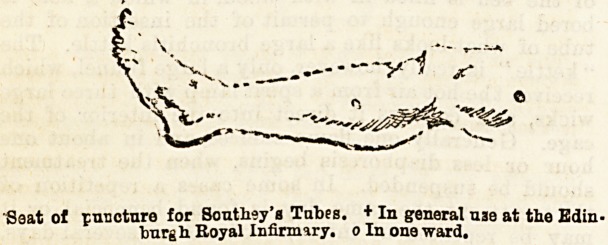# The Treatment of the Dropsy of Bright's Disease

**Published:** 1892-10-01

**Authors:** 


### ROYAL INFIRMARY, EDINBURGH.

### The Treatment of the Dropsy of Bright's Disease.

Dropsy occurs in the acute as well as in the more or less chronic forms of Bright's Disease. The treatment of the condition, as carried out in the Royal Infirmary, Edinburgh, has been grouped under certain headings for the sake of perspicuity and simplicity. I. Remedies used, which act on (*a*) the bowels, (*b*) the skin, (*c*) the heart, (*d*) the kidneys. II. Instrumental methods of treatment. III. Dietetic principles.

I. A. *The Bowels.*—Hydragogue cathartics are very largely used in the Royal Infirmary, and of all, compound jalap powder in 20 to 30 grain doses is the most universal favourite. Professor Grainger Stewart considers that a few grains of calomel or blue pill often increase its efficacy. Elaterium is sometimes used, but with caution.

Concentrated solutions of saline purgatives, such as sulphate of magnesium, the acid citrate of potash and kindred salts are also much given in certain cases in preference to jalap. "Heniy's solution" is the popular purgative in all the wards, medical and surgical, and is largely employed in cases of Bright's disease. Its formula is—

℞ magnesii sulphatis, ʒiv; acidi sulphurici diluti, ʒj; aquam, ad ℥j.

Sig.—One-half or the whole as required.

Such remedies if given warm have their efficacy much increased. Nausea is sometimes found to follow the administration of jalap, and also salines; generally, however, one or other can be borne.

B. *The Skin.*—By means of the skin, with its sweat glands, the bowels are aided to a remarkable extent in getting rid of the excessive amount of fluid in the subcutaneous tissues and various cavities. The skin is stimulated by—

(1) *Medicinal Agents.*—Jaborandi and its active principle, pilocarpine, are employed in the emergency of a uræmic attack; but where there is no uræmia —merely dropsy—the practice in the different medical wards varies considerably. In several wards a 1-6th grain of nitrate of pilocarpine is given hypodermically, or sometimes 1-12th grain along with the hot-air bath. In others it is looked upon with some dread, as it is believed that a toxic action of its own—probably carbonic acid gas poisoning—may be added, but the internal use of jaborandi or pilocarpine in moderate doses is very frequently prescribed. Nitro-glycerine has been found most beneficial, and is very largely employed along with the hot-air bath. Professor Fraser prefers nitrite of sodium.

(2) *Baths.*—Perhaps of even as great importance as the medicinal stimulation of the sudoriferous glands is that accomplished by means of baths, and specially the hot-air bath, although little employed in several wards owing to risks of pulmonary congestion, &c., alleged against it, it is more or less extensively used in others, chiefly in acute Bright's disease, and of course especially where uræmia has supervened. The method of application in the Royal Infirmary here is as follows: A large cage is placed over the patient extending from the

The Hot Air Bath, showiıg the arrangement of the lamp and bed cage.

neck ownwards, and must be long enough to cover over the whole body (see diagram). The patient is rolled up in a blanket, the nightshirt being removed, and a mackintosh sheet and then blankets are placed over the cage and tucked in all round so as to prevent the entrance of cold, and, with the same object in view, are fastened as closely as is compatible with comfort round the patient's neck. The end of the cage towards the foot of the bed is filled in with wood, in which a hole is bored large enough to permit of the insertion of the tube of what looks like a large bronchitis kettle. The "kettle" is really, however, only a large funnel, which receives the hot air from a spirit lamp with three large wicks, and conveys it direct into the interior of the cage. Generally one flame suffices, and in about one hour or less diaphoresis begins, when the treatment should be suspended. In some cases a repetition of the treatment the same day is found beneficial, or it may be repeated at intervals of one or several days. Care is taken to stimulate the heart when necessary with brandy or tincture of strophanthus (or its active principle strophanthin), and the temperature by means of the thermometer in the mouth, the pulse by placing the finger on the carotid, and the respirations, are all watched. Faintness, increase of pulse or respirations, especially if the rhythm become irregular, are indications for discontinuing the bath. Very frequently diaphoretics as already stated are given along with the hot air bath. The simpler measures of placing hot bottles round the patient who is enveloped in blankets, or of wrapping him in a blanket wrung out of hot water, are often employed. Sometimes vapour baths are given with an apparatus similar to what has been already described, only steam is introduced into the cage in place of hot air.

C. *The Heart.*—In acute Bright's disease, where cardiac stimulants or tonics are requisite, alcohol, citrate of caffein, strophanthus, or digitalis is employed. Professor Fraser does not consider that digitalis has either a specially beneficial, nor, on the other hand, has it a specially prejudicial effect. In such cases, in most of the wards, the other cardiac tonics are preferred to digitalis. In chronic Bright's disease, especially where the heart requires it, digitalis is very generally given, and in several wards the infusion in 40 to 80 minim doses is preferred. Professor Grainger Stewart recommends the combination of the tincture of the perchloride of iron with digitalis; and sometimes prescribes squills and carbonate of ammonia with digitalis leaf in the form of a pill.

D. *The Kidneys.*—In acute Bright's disease rest for the inflamed organs is the principle which actuates treatment. Professor Grainger Stewart approves of giving diluent drinks of barley water or milk, along with diaphoretic treatment, in order to flush the organ when its congested condition has been relieved by the dilatation of cutaneous vessels. Dry cupping over the loins is universally adopted, and in some cases, where other measures fail, moist cupping is resorted to. In chronic Bright's Disease, acetate of potash in 15-grain doses—the combination of tincture of the perchloride of iron with spirit of nitrous ether and other well-known diuretics are popular. Dr. Brakenridge gives citrate of caffein very extensively in cases of Bright's disease after the more acute stage is over, and finds the albumen and tube casts very rapidly diminish, while the urea correspondingly increases.

II. *Instrumental Methods of Treatment.*—The favourite topic treatment is by tapping, and Southey's tubes are preferred to multiple needle punctures or incisions, but the legs, for instance, are never so drained unless the anasarca is great. With proper antiseptic precautions the general belief is that the procedure is a perfectly safe one, the risks of erysipelas produced by the irritation of the tube being very slight, and in support of this in several cliniques not a single case has gone wrong. The capillary tubes and Southey's trocar and canula are washed with carbolic and then boracic lotions, as carbolic would coagulate the albumen in the dropsical fluid, and the limb to be operated on is also washed with boracic. The part of the limb selected for puncture is sometimes the dorsum of the foot, or just below the external malleolus or the outer aspect of calf or thigh (see diagram).

Seat of puncture for Southey's Tubes. + In general use at the Edinburgh Royal Infirmary. o In one ward.

The greatest care should be taken in sealing up the punctures after withdrawing the canula, otherwise they will leak continuously, and the discharge may cause sloughing, or at least irritation of the already devitalised skin. Collodion or adhesive plaster may be used, but a compress as applied by Dr. Wyllie acts remarkably well. It consists of a pad of lint which is placed over the puncture, and two strips of adhesive plaster are then applied over the pad crosswise, and attached to the skin on either side. If the ends of the strips are fastened down when the skin is drawn up towards the site of puncture the elasticity of the latter keeps the plaster tightly applied over the pad, thus giving sufficient pressure to prevent any leakage. After tapping the limbs bandaging from below upwards is frequently adopted, and sometimes this procedure is carried out where the anasarca is not sufficiently great to warrant the use of Southey's tubes. For the scrotum puncturing with a needle in several places is carried out where requisite, with the usual antiseptic precautions, and antiseptic absorbent wool of a non-irritant character is used for soaking up the discharge. In no ward is the treatment by incision carried out as routine practice, though in some exceptional cases it has been tried.

In hydrothorax of Bright's disease the almost universal custom is to tap, especially if the patient is dyspnœic, whereas in ascites there is a good deal of difference of opinion. Several of the staff hold that as often as the patient is tapped the peritoneal cavity fills up again and the greater will be the drain on the system. In other wards, and especially on the University side of the Hospital, tapping is more freely carried out. For paracentesis thoracis, Potain's aspirator is used; for paracentesis abdominis; sometimes the aspirator, often a larger Southey's tube than that used for the limbs is employed.

Special attention is paid to antiseptic precautions. The tubing attached to the Southey's canula should dip in the first instance into some boracic solution. Where the tubing is liable to pressure as at the edge of the bed a safety pin passed through the lower sheet on either side of the tube and about half-an-inch from it, forms a protecting channel for it. When draining the peritoneal cavity a broad "double roller" flannel bandage should be passed round the front of the abdomen and the two roller ends crossed behind, so that the patient, if able, can himself keep up and gradually increase the pressure. Where a Southey's tube is used the draining may go on for several hours, and the bandage can be fixed with pins and tightened from time to time. After tapping the thorax or abdomen a broad bandage is always firmly applied.

III. *Dietetic Principles.*—In most of the wards milk diet has much favour, especially in acute Bright's disease; and in chronic cases a check is, of course, placed upon the fluid drinking capacity of the patients. A strictly dry diet is much advocated by Dr. Wyllie for a strong patient the subject of acute Bright's disease, consisting of water, arrowroot, with a measured pint of milk as a beverage. This regimen cannot, however, be persisted with for over two days as it becomes so soon unbearable. Great stress is laid in all the wards on the value of peptones, which being easily digested, or, rather, already digested, rapidly improve the condition of body and blood.

## Figures and Tables

**Figure f1:**
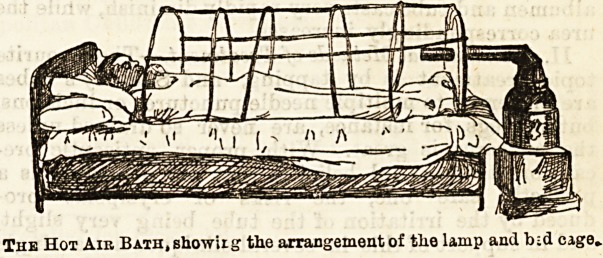


**Figure f2:**